# Gastro-Enteritis

**Published:** 1874-05

**Authors:** 


NOTES OF PRACTICE IN ROOSEVELT HOSPITAL.—*Gastro-Enteritis.*—Some cases of this character, which were characterized by loose passages from the bowels, from two to four in number daily, and more or less of gastric disturbance, have, after resisting other remedial measures employed, rapidly improved by use of a pill composed of nitrate of silver $\frac{1}{4}$ of a grain, and sulphate of morphia $\frac{1}{8}$ of a grain three times a day.

